# National Medical Convention

**Published:** 1847-08

**Authors:** 


### EDITORIAL DEPARTMENT.

---

*National Medical Convention.*—[The following remarks were written for the June No. of the Journal, and have hitherto been excluded by the press of other matter. We take this occasion to state, that we shall soon redeem our promise to furnish our readers with the code of Medical Ethics adopted by the Convention.]

The number of delegates which were assembled at the convention, and the fact that nearly all the states of the Union were represented, afforded sufficient evidence that a desire for union and organization prevails among the profession throughout the country: if farther proof were required, the harmony and unanimity which pervaded the convention, and characterised all its actions, abundantly demonstrate it. It may be questioned if the deliberations of so large a body upon subjects of deep interest to each individual member, ever exemplified a better spirit. Assuredly we may refer to that meeting, as well as to the convention which preceded it, as showing conclusively that the medical profession (of this country at least) are not so prone to disagreement and wrangling as is often sneeringly insinuated. It was remarked by a spectator, that he had attended many ecclesiastical assemblages, but he had never observed, even on those occassions, such entire absence of bitterness of controversy, and such scrupulous observance of courtesy and decorum, as in the present instance. Nothing, in fact, occurred to mar the dignity of the convention, or diminish the satisfaction which every member appeared to feel in being present. If we were to indulge any criticism it would be to take some exceptions to the disposition manifested by a few to occupy too exclusively the time and attention of the convention in the discussion of every subject. If we could flatter ourselves that our advice would reach the persons obnoxious to this remark, and that it would exert any influence provided it did reach them, we should be tempted to counsel them to endeavor to restrain hereafter that *cacoethes loquendi* to which they are prone on such occasions. We would enjoin this, not only because their strenuous efforts to anticipate others displaces those whom it would have been more acceptable should have been oftener heard, but, also, because the result is, that they failed to secure for themselves that consideration which they so much desired, and which they might have obtained, if they had exercised the art of keeping silence, as well as the art of speaking well.

The most important of the special acts of the convention was the recommendation to medical schools to extend the term of lectures from four to six months. As to the great advantage which will accrue to medical instruction from adopting this course, we have no doubts. The present period is too limited for teachers to do justice to their several departments, without crowding into a given space, a greater amount of labor for the pupil than he can perform. The number of lectures now given daily at most medical institutions is greater than is compatible with proper attention and benefit on the part of the student, and, to some extent, also, with due devotedness on the part of the instructor. We believe such is the view generally entertained by those connected with medical institutions, and we do not doubt that they will be inclined to extend the term of lectures in conformity with the recommendation of the convention. The necessity of all or nearly all the schools uniting in order to effect this change is obvious. It is not to be expected that a few will consent to extend their terms unless sustained by a similar action by the majority. It is natural and proper to look to some of the older metropolitan schools to take the lead in this matter. The* University of Pennsylvania, the College of Physicians and Surgeons in New-York, and the Boston school. for examples, should be among the first to move. We hope each or all these institutions will soon announce an intention to follow the recommendation of the convention, and we are persuaded that other institutions will at once, on speedily follow their example. It has been suggested, and with some force, that to extend the lecture term for the ensuing season to six months, might be considered to be acting in bad faith to those students who have attended one course of lectures under the present arrangement. In order to meet this objection, and to avoid the difficulties of a sudden change, we would suggest that for the next session, the term be extended to five months, notice being given in forthcoming circulars that after the ensuing session the sixth month will be added.

It had been supposed by some that one of the subjects which came before the convention, viz:—the proposition to dissever teaching and licensing, would occasion much excited discussion, if not be productive of discord. There appeared, however, to be pretty general unanimity in deferring action upon this subject until it had received farther consideration by the profession at large, all being agreed that the object of the convention was not to *revolutionize* but to *reform*, and that important changes should not be recommended without due deliberation. In justice to those who expressed views in advocacy of the proposition referred to, it should be stated, that they severally disclaimed any wish to depreciate medical schools, or to derogate from their importance to the medical student. The proposition,as enunciated by its advocates in the convention, is not to deprive institutions of their prerogative of conferring the doctoral degree, but to institute boards of examiners in each state, consisting in part of those who are, and in part of those who are *not* connected with schools, whose approval of qualifications shall be necessary to confer a license to practice. We will not now stop to discuss the propriety or practicability of [illegible] is

*By reference to another page, it will be seen that the Univ. of Pa. has taken [illegible] lead.

scheme. It was by no means exclusively supported by those not connected with schools, and who might be suspected of hostility to them, but was advocated by several who hold distinguished positions as public medical teachers. The few who, from local circumstances, or personal considerations, may have indulged hopes that a spirit of animosity toward medical institutions would ensure discord if it did not declare itself in the action taken by the convention on the subject, were egregiously mistaken. If members were present who entertained such feelings their expression was restrained by the moral influence which the character of the convention, and the general tone of sentiment which prevailed, could not fail to exert.

The important ends attained by the convention are not to be measured by its special acts. A spirit and energy have been developed and diffused by these *reunions* which will not soon be quenched, and which will be productive of incalculable benefit both to the science and the profession. Their influence may already be observed in the new life which has been infused into local associations, and state conventions. We have expressed on a previous occasion the opinion that they would constitute an epoch in the history of medicine in this country. We repeat the remark with a still stronger conviction that the prediction will be verified. In order for the profession of this country to become all that it is capable of becoming, and to accomplish all that it could reasonably expect to accomplish, it is only necessary to feel and act unitedly. There is no body of men in the country that can exert greater influence and power to effect any objects than the Medical Profession. Let there be unanimity as to what will really prove advantageous, and united zeal in the endeavor to procure what is desired, and success is certain. In these remarks we do not refer solely to objects of a scientific nature, but to those which involve the sentiment of community, or the acts of Legislation. The moral, and, we will add, (but in no unworthy sense) the political power, which, as a profession, acting unitedly, we may exert, must almost necessarily be irresistable. We have only to be persuaded in our own minds what ends it will be the part of wisdom to seek for, and to act perseveringly, and in concert for their attainment—and they will be accomplished. We conceive that the correctness of this statement might be rendered as clear and convincing as speculative reasoning can establish any truth of a similar nature.

The institution of the *National Medical Association* we deem the most important of the acts of the convention. We now have a permanent organization, by means of which all may co-operate in furthering scientific objects, and in promoting the interests of the profession. Having recently considered some of the advantages which, in our estimation, may be derived from such an association, we will not, at present, offer any farther remarks on this subject. With respect to the details of the organization, upon which there was some discrepancy of opinion, we may present our views hereafter. Should it be found that improvements in the constitution adopted by the convention are desirable, they can at any time be effected, and inquiry with respect to this point should engage the attention of the profession.

In conclusion, we think the profession have cause for gratulation that the movement for *union and organization* has progressed thus far in so satisfactory a manner. Let the same zeal and harmony continue, and we cannot but hope, and believe, that results will be attained which will be highly conducive to the interests of medical science, the medical profession, and of humanity.

